# Repair of a Supracristal Ventricular Septal Defect in an Adult

**DOI:** 10.7759/cureus.10752

**Published:** 2020-10-01

**Authors:** Conrad Gray, John Pirris, Adrienne Warrick, Saurin Shah

**Affiliations:** 1 Anesthesiology, University of Florida College of Medicine, Jacksonville, USA; 2 Cardiothoracic Surgery, University of Florida College of Medicine, Jacksonville, USA; 3 Anesthesiology, University of Florida, Jacksonville, USA

**Keywords:** ventricular septal defects, supracristal ventricular septal defects, pulmonary hypertension, nitric oxide, aortic insufficiency, transesophageal echocardiogram

## Abstract

Ventricular septal defects (VSDs) are the most common congenital cardiac abnormalities occurring in five out of every 1000 births. Supracristal VSDs (located above the crista supraventicularis) are very rare and comprise only 2%-3% of all VSDs. Many VSDs close spontaneously during childhood; however, a substantial portion may not and are present in adulthood with a myriad of symptoms. We describe the management of a complex case of an adult patient with a supracristal VSD and resultant severe aortic insufficiency (AI) in the perioperative setting.

## Introduction

Supracristal ventricular septal defects (VSDs) are typically large lesions and, unlike other areas of the septum, do not decrease in size or spontaneously close. While very infrequent, they can lead to prolapse of the right cusp of the aortic valve (AV) predisposing affected patients to aortic insufficiency (AI) via the venturi effect. Aortic valve prolapse (AVP) and AI are absent at birth; however, the risk of developing AVP and AI increases with peak occurrence between the ages of 5 and 10 years. The majority of these lesions are surgically closed early in childhood to avoid or minimize the complications and physiological changes that may develop with AI and VSD shunting. The rare presentation of an adult supracristal VSD presents many challenges in the perioperative setting that are different than other VSDs.

## Case presentation

The patient is a 60-year-old African American male with multiple medical issues presenting with chest pressure, dyspnea on exertion, and progressive bilateral lower extremity edema. Evaluation and work-up of this patient revealed the presence of a supracristal VSD with severe pulmonary hypertension and both right and left ventricular dysfunction with a left ventricular ejection fraction (LVEF) of 35%-40%. Interdisciplinary discussions determined that the patient was not a candidate for a minimally invasive repair of his VSD. The patient was scheduled for open surgical repair by a team consisting of both an adult and a pediatric congenital cardiac surgeon.

The patient was brought to the operating room and a pre-induction radial arterial line placement was performed. After uneventful induction, intubation and central venous line placement, transesophageal echocardiography (TEE) was placed. The TEE revealed the VSD seen best in midesophageal long axis (MELAX) view. Left to right flow through the VSD occurred throughout the cardiac cycle. AI with an eccentric jet directed towards the basal anterior ventricular septum was seen but without evidence of AV leaflet prolapse. In short axis the AV was trileaflet with normal leaflet excursion, possible asymmetric leaflet size (smaller left coronary cusp). Sinus of valsalva was normal and nonaneurysmal.

Midline sternotomy was performed. The patient was heparinized, pericardium was opened and suspended. Aortic cannulation followed by tricaval cannulation (patient had a persistent left superior vena cava) occurred without complications. Cardiopulmonary bypass (CPB) was initiated and cardioplegia was administered antegrade as well as via the coronary ostia after performing an aortotomy. An incision was made in the pulmonary artery to locate and inspect the VSD; after failing to locate the VSD, the aortotomy was extended to reveal the sub-valvular septum and the VSD. After thorough direct visualization and evaluation of the VSD, bovine pericardium was used to repair the defect. Inspection of the AV revealed relatively normal leaflets with fenestrations at the left and right coronary cusp commissures; resuspension of the commissures was performed. After cross clamp release and de-airing maneuvers, initiation of CPB wean and TEE was again performed. The TEE revealed significant severe, central AI. No residual defect was seen at the site of the supracristal VSD. The decision was made to return to CPB and replace the AV with a 25 mm Medtronic Mosaic® tissue valve (Medtronic, Minneapolis, MN, USA). De-airing maneuvers were performed again and the aortic cross clamp was removed in advance of a slow wean off CPB. TEE revealed well-seated bioprosthetic AV, no perivalvular leak, or significant regurgitation. There was significant continued left ventricle (LV) dysfunction and right ventricle (RV) dilation and dysfunction. Intra-aortic balloon pump (IABP) was placed via the right femoral artery under TEE guidance and the patient was started on inhaled nitric oxide at 40 ppm in addition to IV infusions of epinephrine and norepinephrine to support contractility and systemic vascular resistance (SVR). LVEF and SVR improved; TEE exam revealed decreased RV dilation and improved LVEF from pre-IABP function. The patient was successfully weaned from CPB, and at conclusion of surgery the patient was transported to the ICU. While in the ICU, the patient was noted to have progressively increased chest tube output; decision was made to take the patient back to the operating room for re-exploration and any other indicated procedures. The source of bleeding was located and controlled; the patient returned to the ICU post-operatively.

The figures displayed are a sample of the views obtained during intraoperative TEE. Images obtained during AV repair/replacement are often not challenging to obtain and are simply to assess if AI is improved, unchanged, or worse. However, in the setting of a supracristal VSD, this TEE exam was notably more challenging. 

**Figure 1:**
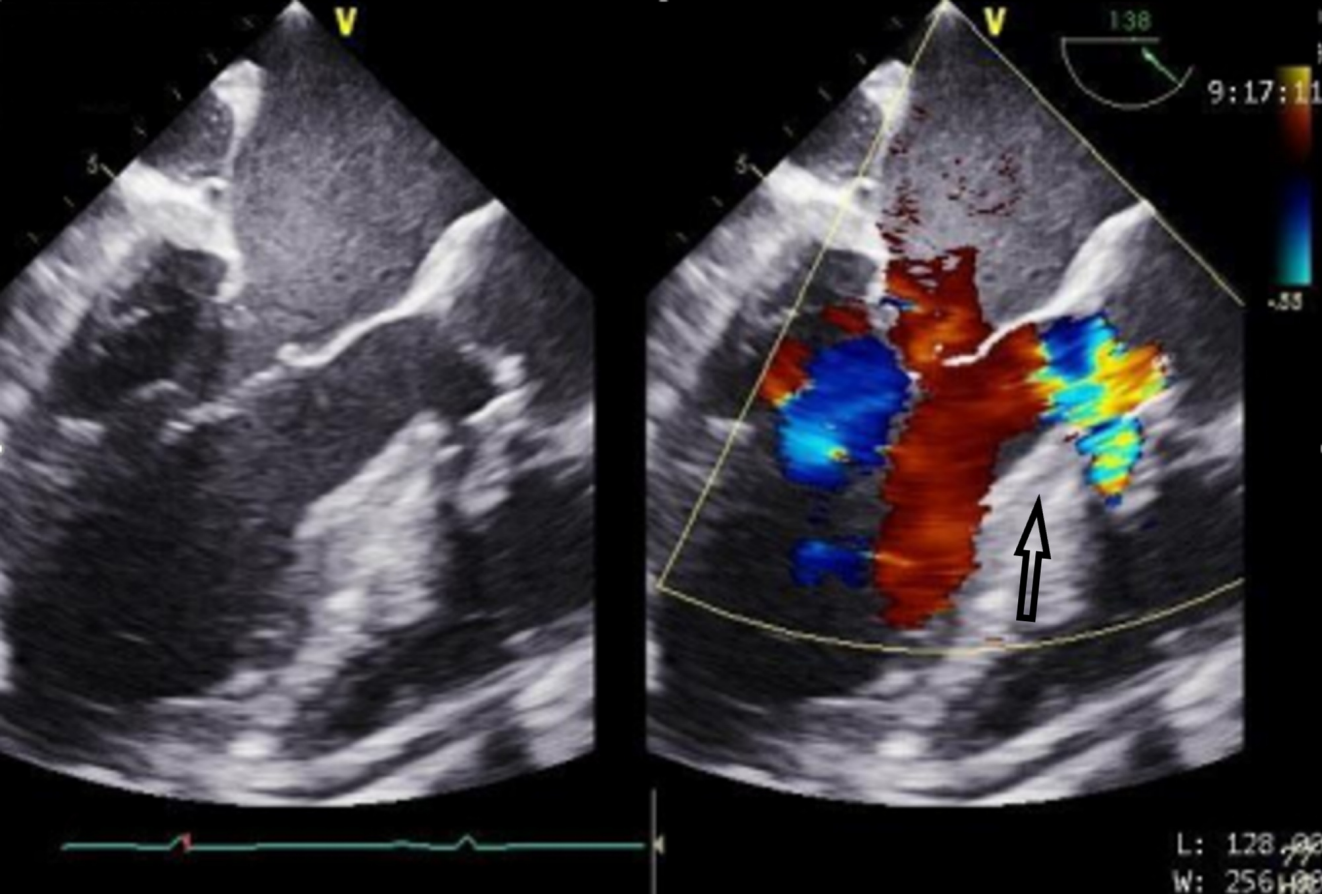
Midesophageal long axis view depicting the left atrium (top) and LV (bottom). Color flow doppler is used on the right figure to demonstrate the severity of AI (arrow). LV, left ventricle; AI, aortic insufficiency

**Figure 2:**
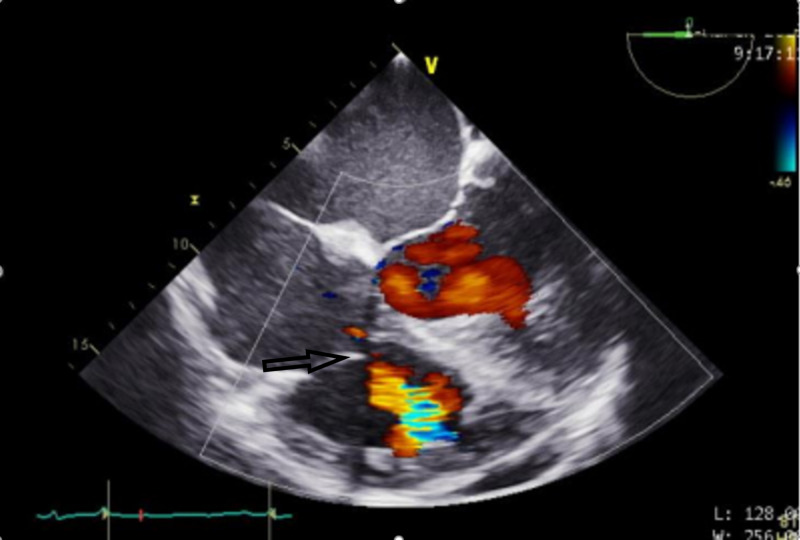
Midesophageal four chamber view. Color flow Doppler is used to show flow through the VSD shown by the arrow. VSD, ventricular septal defect

**Figure 3:**
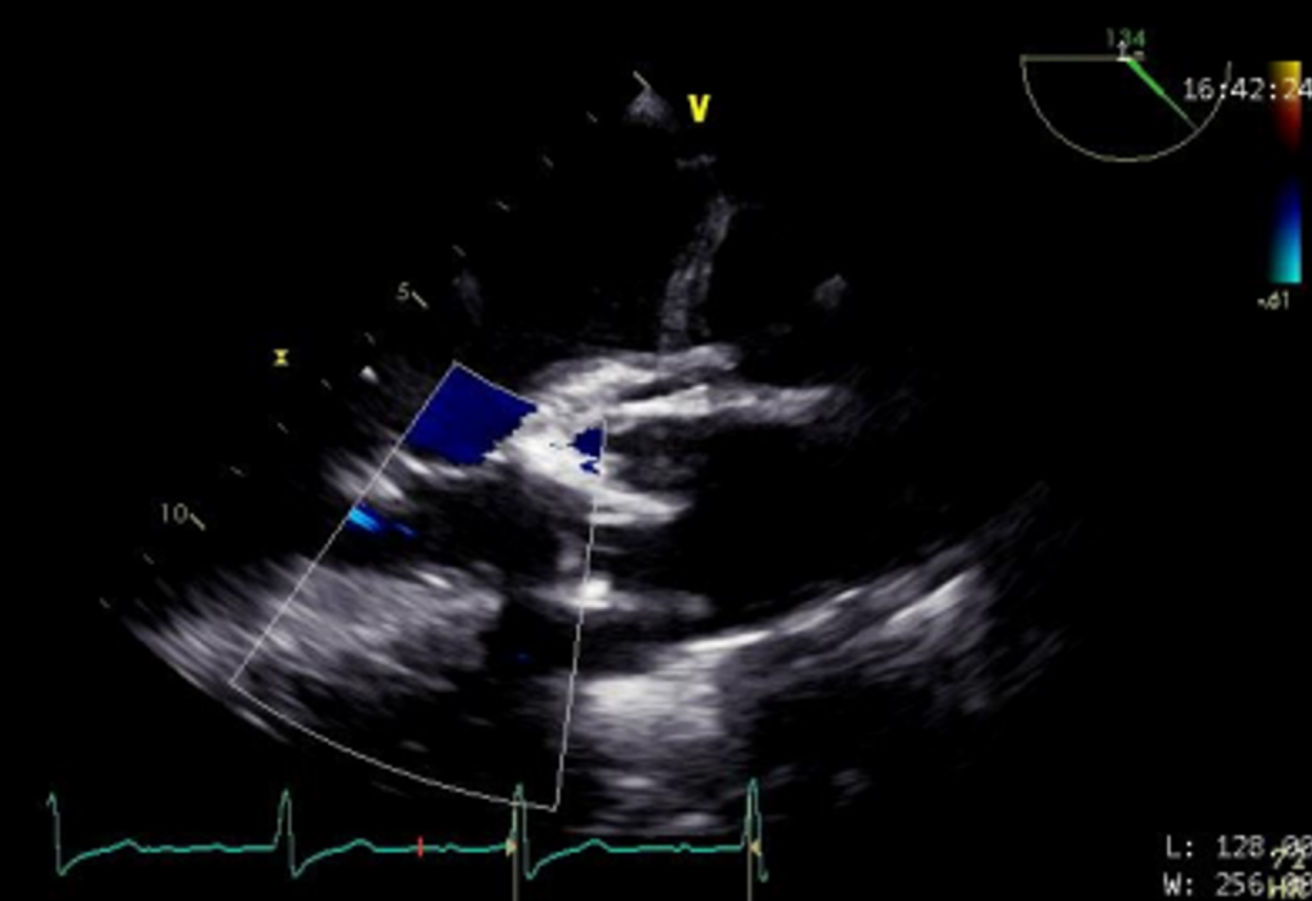
Aortic valve status post repair. TEE is vital after valve repair to assess for improved/unchanged/worsened valvular insufficiency which dictates any further surgical intervention. TEE, transesophageal echocardiography

Figures 1-2 highlight the defects prior to repair. These images are abnormally challenging to obtain due to the complexity of AI in the setting of a supracristal VSD. These images are obtained to guide the surgical plan. Figure 3 highlights the improved aortic sufficiency following repair. 

## Discussion

A supracristal VSD arises from the absence of the subpulmonary muscular infundibulum creating continuity between the aortic and pulmonic valves [1-2]. These are very rare congenital defects that are repaired early in childhood; the presence of these lesions in the adult population is extremely rare and may present with a wide range of symptoms and pathology [3-4]. This case highlights the surgical and perioperative challenges associated with a late presentation/diagnosis of a supracristal VSD in an adult; it demonstrated an attempted repair and resuspension of the AV leading to worsening AI and subsequent AV replacement. The effects of age, size, and other chronic cardiovascular pathology such as atherosclerosis and calcium-related vascular disease may account for the different result in AV repair surgery.

## Conclusions

Literature detailing the pathophysiology of supracristal VSD and associated AI supports repair of the AV as a management option in the pediatric population; however, there is a paucity of literature noting AV repair success in the adult population. The management of this pathophysiology of adults requires a multidisciplinary approach involving both adult/pediatric and skilled echocardiographers to achieve the best possible outcomes. Additionally, further investigation into the adjunct use of inhaled nitric oxide and an IABP may help in the future management of these patients.

## References

[1] Miller LR, Nemeth M, Flamm SD, Sung C, Stainback RF (2006). Supracristal ventricular septal defect. Tex Heart Inst J.

[2] Egbe AC, Poterucha JT, Dearani JA, Warnes CA (2015). Supracristal ventricular septal defect in adults: is it time for a paradigm shift?. Int J Cardiol.

[3] Yacoub MH, Khan H, Stavri G, Shinebourne E, Radley-Smith R (1997). Anatomic correction of the syndrome of prolapsing right coronary aortic cusp, dilatation of the sinus of Valsalva, and ventricular septal defect. J Thorac Cardiovasc Surg.

[4] Mongeon FP, Burkhart H, Ammash N, Dearani J, Li Z, Warnes C, Connolly H (2010). Indications and outcomes of surgical closure of ventricular septal defect in adults. JACC Cardiovas Interv.

